# Superior mesenteric artery syndrome in a 26-year-old male presenting as acute pancreatitis and portal venous gas: a case report and review of the literature

**DOI:** 10.1186/s13256-026-05934-y

**Published:** 2026-03-04

**Authors:** Witchapas Punyanirun, Pakkapol Sukhvibul, Issaree Laopeamthong, Wisit Kasetsermwiriya, Suphakarn Techapongsatorn

**Affiliations:** Department of Surgery, Faculty of Medicine Vajira Hospital, Navamindradhiraj University, Bangkok, Thailand

**Keywords:** Superior mesenteric artery syndrome, Acute pancreatitis, Portal venous gas, Gastric emphysema, Duodenal obstruction, Nonoperative management

## Abstract

**Background:**

Superior mesenteric artery syndrome is a rare form of duodenal obstruction that usually requires nonoperative management. While individual complications such as acute pancreatitis, portal venous gas, and gastric emphysema have been reported, the simultaneous occurrence of this triad in a single patient with superior mesenteric artery syndrome has never been documented. This case is novel as it highlights the successful nonoperative management of superior mesenteric artery syndrome despite the presence of these severe, potentially life-threatening complications.

**Case presentation:**

A 26-year-old Asian male with a constitutional low body mass index of 14.53 kg/m^2^ presented with abrupt epigastric pain and bilious vomiting. Investigations revealed a diagnosis of superior mesenteric artery syndrome complicated by acute pancreatitis, portal venous gas, and gastric emphysema. The patient was treated nonoperatively with intensive care support, including nasogastric decompression and total parenteral nutrition. The interventions led to a significant reduction in gastric output, resolution of acute kidney injury and pain, and subsequent removal of the nasogastric tube. The patient was discharged well and showed no signs of obstruction at the 9-month follow-up, having gained weight.

**Conclusions:**

This case demonstrates that the presence of portal venous gas and gastric emphysema in superior mesenteric artery syndrome, which typically raises suspicion for gastrointestinal necrosis, can be successfully managed conservatively if clinical signs of perforation or sepsis are absent. Clinicians should maintain a high index of suspicion for the complex presentations of superior mesenteric artery syndrome and prioritize aggressive nonoperative nutritional support, as it can resolve obstruction and preclude the need for surgery even in the setting of severe multi-systemic complications.

## Background

Superior mesenteric artery (SMA) syndrome, also known as Wilkie’s syndrome, is a rare acquired disorder characterized by the compression of the third part of the duodenum between the abdominal aorta and the overlying superior mesenteric artery [1]. Anatomically, the condition results from the narrowing of the aortomesenteric angle, typically maintained by the aortomesenteric fat pad [2].

Various conditions that cause the depletion of this fat pad include rapid weight loss, severe physiological stress, and anatomical variations such as a high insertion of the ligament of Treitz [3]. The reported prevalence is between 0.1 and 0.3%, with a higher incidence in younger females and underweight individuals [2–4].

Diagnosis is confirmed using computed tomography (CT) scan, which allows for the measurement of the aortomesenteric angle (typically < 22°) and aortomesenteric distance (< 8 mm) [5]. Treatment typically begins with nonoperative management, including gastric decompression and nutritional support, which has a success rate of over 70% [6]. Surgical intervention, such as duodenojejunostomy, is reserved for cases where conservative treatment fails or complications arise [7].

While complications such as acute pancreatitis or pneumatosis have been described individually, the simultaneous occurrence of acute pancreatitis, portal venous gas, and gastric emphysema in a single patient is exceptionally rare. This case report describes the successful nonoperative management of a patient presenting with this unique and severe triad of complications.

## Case presentation

A 26-year-old Asian male presented to the emergency department with the abrupt onset of severe, stabbing epigastric pain radiating to his back. At 3 days prior to admission, he experienced vague epigastric discomfort and postprandial bilious vomiting. He reported no significant medical or surgical history, and denied using weight reduction pills, having eating disorders, smoking, or heavy alcohol use. His baseline weight was 42 kg, height 170 cm, BMI 14.53 kg/m^2^; family confirmed he had been this weight chronically without intentional loss.

Physical examination revealed a fatigued patient (performance status 3) who was febrile (body temperature 38 °C) and tachycardic (pulse rate 110 beats per minute). Other vital signs were within normal limits. Mild epigastric tenderness was noted without signs of peritonitis, and a succussion splash was positive.

**Fig. 1:**
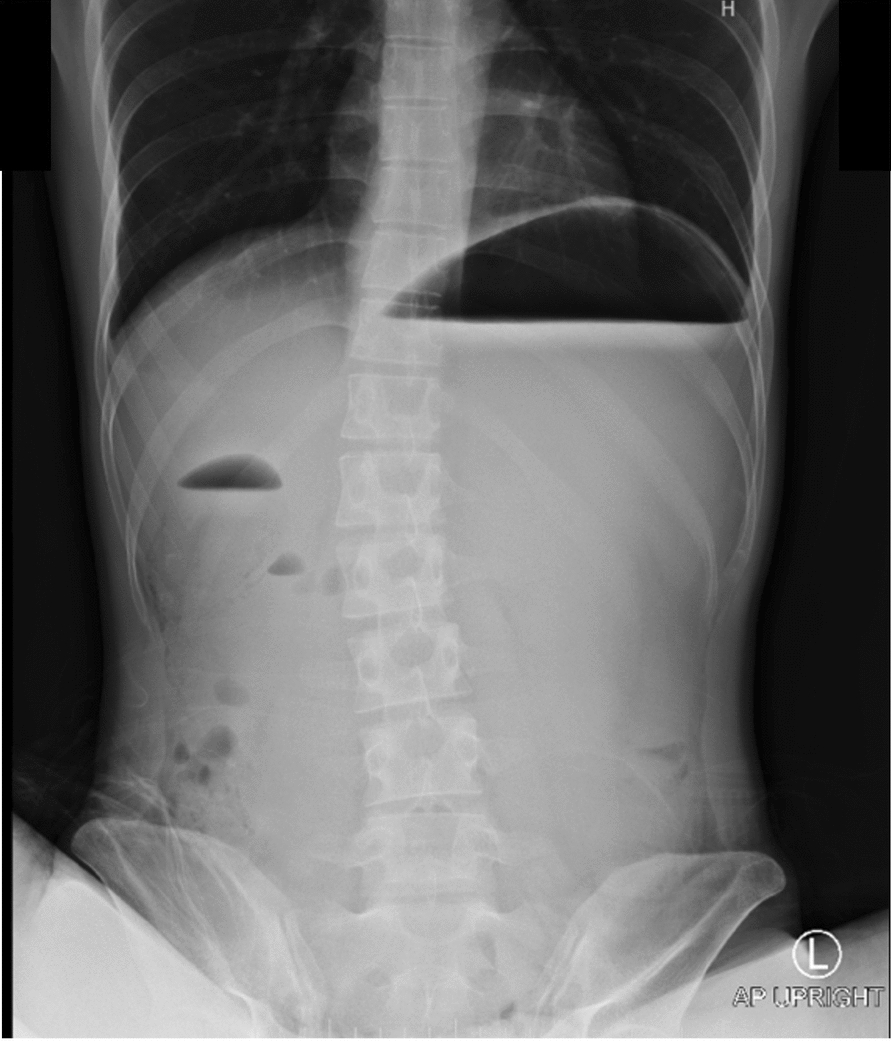
Plain upright abdominal radiograph showing dilated stomach and duodenum

Initial investigations were notable for leukocytosis (16,030 cells/μL), acute kidney injury (Creatinine 3.01 mg/dL), and lipasemia (1964 U/L). Based on the characteristic abdominal pain and elevated lipase levels (greater than three times the upper limit of normal), a diagnosis of acute pancreatitis was made according to the Revised Atlanta Classification. Initial plain abdominal radiography showed marked dilatation of the stomach and proximal duodenum (Fig. 1). A nasogastric tube was placed, yielding 1500 ml of bilious content.

**Fig. 2:**
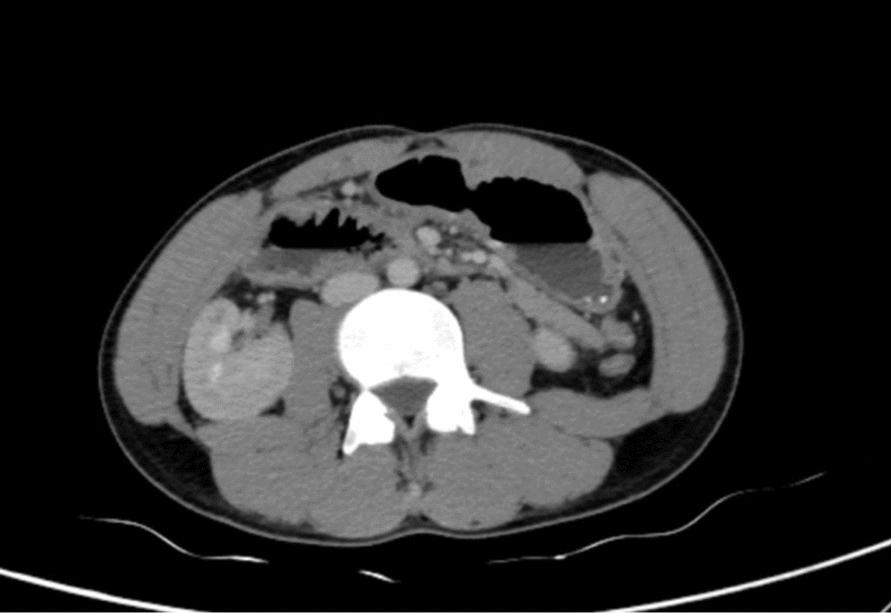
Upstream dilatation beginning at third portion of the duodenum

**Fig. 3:**
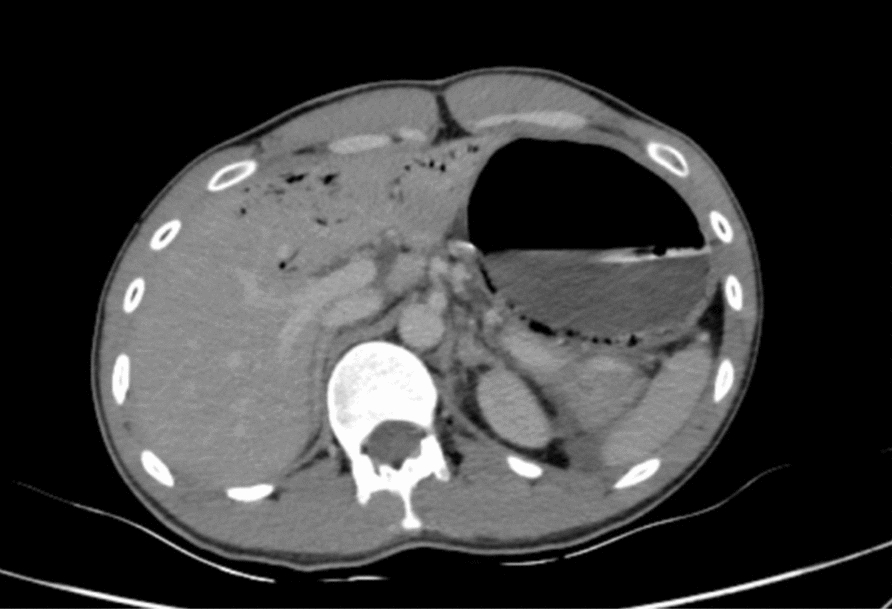
Axial computed tomography showing gastric emphysema and portal venous gas

**Fig. 4:**
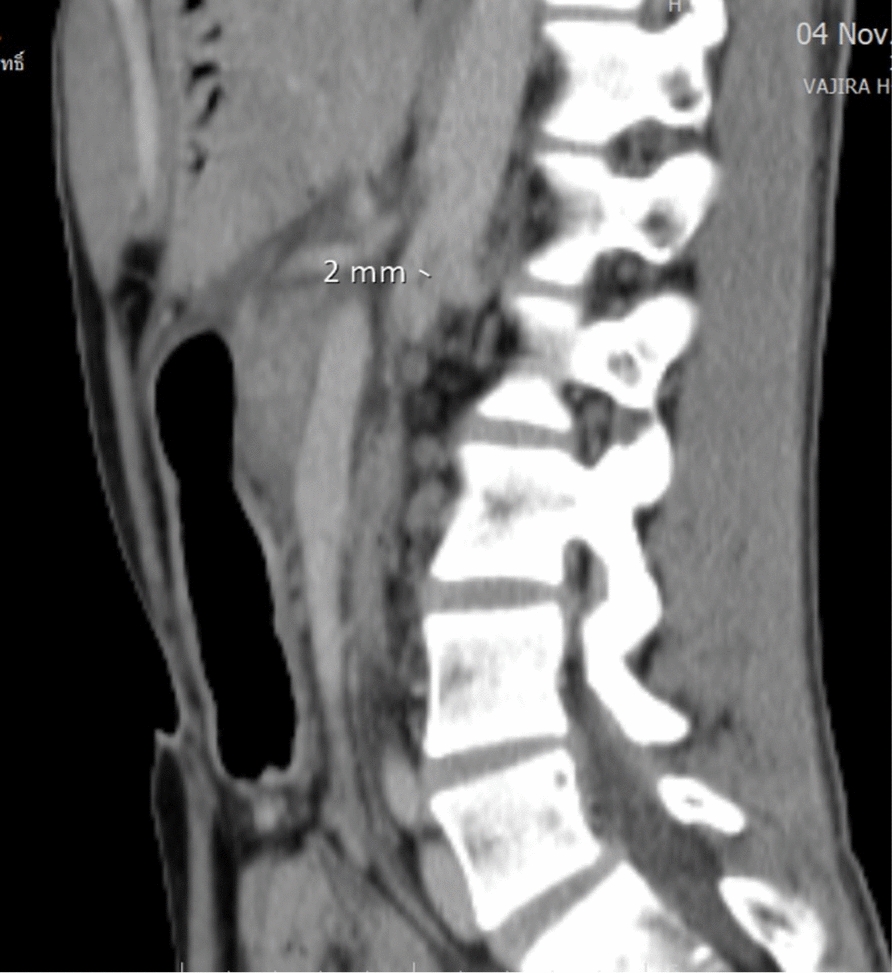
Aortomesenteric distance of 2 mm

**Fig. 5:**
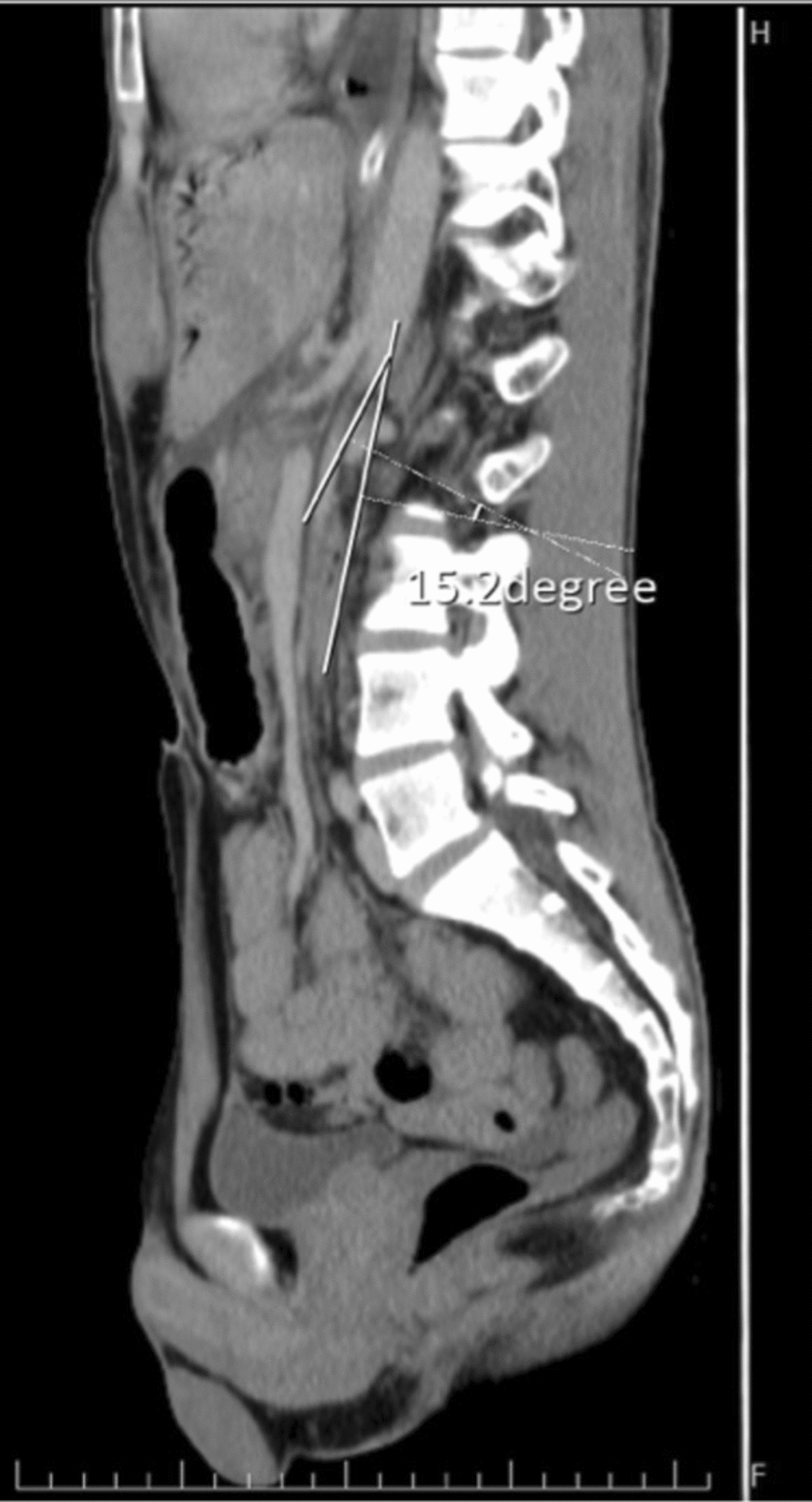
Aortomesenteric angle of 15.2°

Abdominal CT scan revealed marked gastroduodenal dilatation with an abrupt cutoff at the third portion of the duodenum (Fig. 2), along with the concerning findings of linear intramural gas within the gastric wall (gastric emphysema) and branching linear lucencies extending to the periphery of the liver, consistent with portal venous gas (Fig. 3). The CT also demonstrated diffuse enlargement of the pancreas with peripancreatic stranding, consistent with interstitial edematous pancreatitis. No pancreatic necrosis or collections were identified. Importantly, there were no signs of gastrointestinal ischemia or perforation. The CT confirmed SMA syndrome, demonstrating a severely narrowed aortomesenteric angle of 15° and an aortomesenteric distance of 8 mm (Fig. 4, 5).

The diagnosis was superior mesenteric artery syndrome complicated by acute pancreatitis. After excluding other common etiologies (gallstones, alcohol, hypertriglyceridemia, drugs), the pancreatitis was concluded to be secondary to duodenal obstruction. Given the patient’s stable condition and absence of peritoneal signs, the portal venous gas and gastric emphysema were attributed to marked bowel dilatation rather than necrosis, leading to a decision for close observation.

The patient was admitted to the ICU and managed nonoperatively with intravenous fluids, empirical antibiotics, prokinetics, and total parenteral nutrition (TPN). Over the first week, his condition gradually improved: creatinine normalized with good urine output, and abdominal pain resolved. Initial plans for nasojejunal tube insertion were postponed when the patient required intubation for transient volume overload. Following diuresis and extubation, the nasogastric tube output drastically decreased from 1000 ml/day to 200 ml/day.

**Fig. 6:**
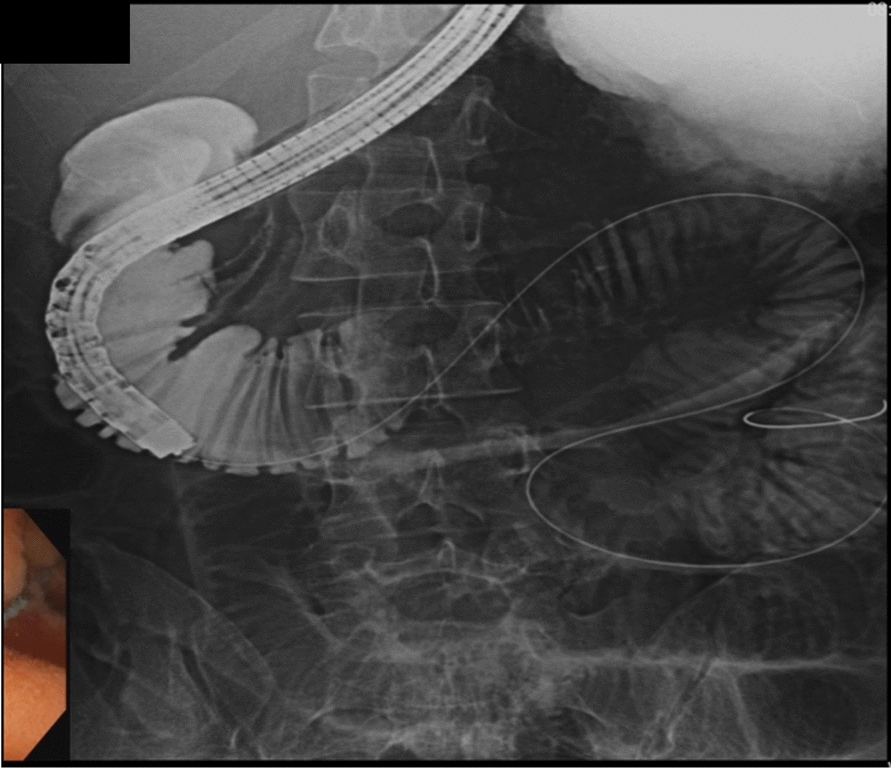
Contrast enterogram at 2 weeks postadmission

**Fig. 7:**
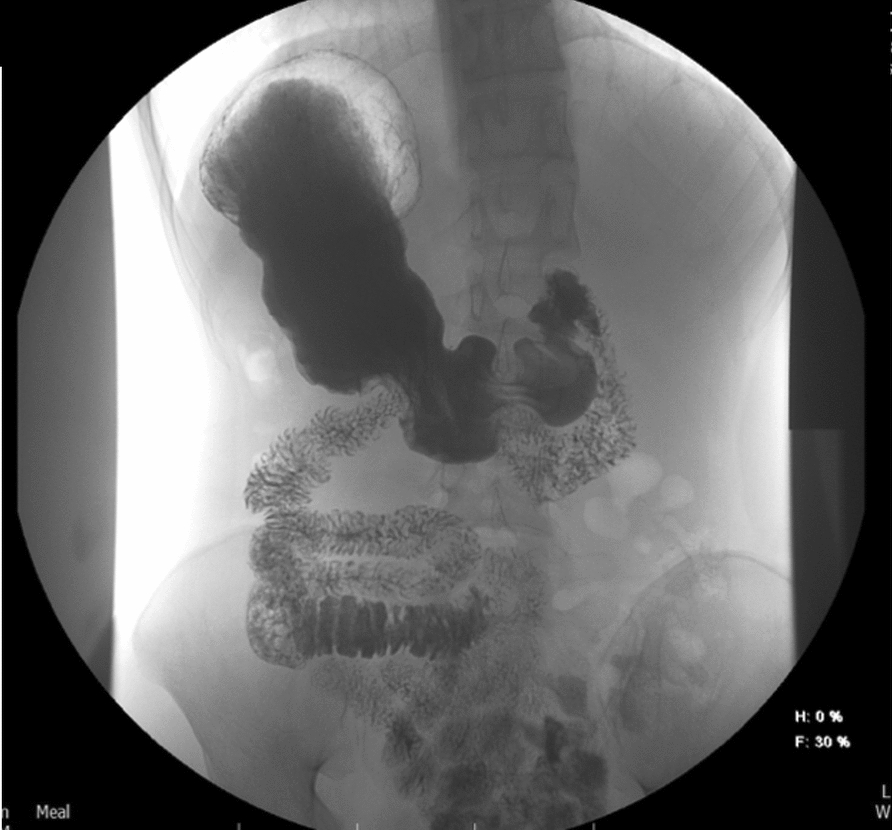
Small bowel follow-through at 1 month postdischarge

At 2 weeks postadmission, an esophagogastroduodenoscopy with contrast enterogram was performed (Fig. 6), showing no mechanical obstruction and only slightly delayed contrast passage. The nasogastric tube was removed, and a liquid diet was started, which the patient tolerated. He was discharged weighing 42 kg. Subsequent small bowel follow-through at 1 month (Fig. 7) confirmed the resolution of obstruction, allowing him to resume a regular diet. At the 9-month follow-up, the patient was doing well and had gained weight (45.5 kg).

## Discussion

This case report details the successful nonoperative management of a highly complex presentation of SMA syndrome, marked by the concurrent presence of acute pancreatitis, portal venous gas, and gastric emphysema. Our case report was the first to report concomitant acute pancreatitis, portal venous gas, and gastric emphysema following SMA syndrome. There have been reports of these individual complications but none with all of these complications in one patient.

The underlying cause of SMA syndrome in our patient appears to be a constitutional factor, as he had a chronically low BMI without recent rapid weight loss. The definitive radiological confirmation of the narrowed aortomesenteric angle and distance supported the diagnosis.

The most notable aspect is the combination of severe complications. Acute pancreatitis is a rare consequence of duodenal obstruction in SMA syndrome, hypothesized to be caused by increased pancreatic duct pressure or retrograde bile reflux [8–10]. It is crucial to recognize that the acute pancreatitis in this patient was secondary to the biliary/pancreatic ductal hypertension caused by the duodenal obstruction. Therefore, the primary therapeutic goal was the management of the SMA syndrome via decompression, rather than solely focusing on standard pancreatitis protocols. Relief of the obstruction facilitates the resolution of the pancreatic inflammation.

The findings of portal venous gas and gastric emphysema typically signify bowel ischemia or necrosis and often mandate immediate surgical intervention. However, reports suggest that in the absence of clinical signs of sepsis, peritonitis, or severe hemodynamic instability, these signs can result from increased intraluminal pressure or marked bowel wall distension, allowing for conservative management [11, 12]. Differential diagnoses for intramural gas include Pneumatosis Cystoides Intestinalis (PCI). However, PCI typically presents with cystic air collections in the small or large bowel. In our case, the CT findings of linear gas distribution restricted to the gastric wall, in the setting of massive gastric dilatation, strongly supported a diagnosis of gastric emphysema secondary to high intraluminal pressure rather than PCI.

Our decision to proceed with nonoperative care—relying on close observation and aggressive decompression/nutrition—was vindicated by the patient’s recovery. The nonoperative approach successfully restored the patient’s nutritional status via TPN, which is thought to increase the retroperitoneal fat pad, thereby widening the aortomesenteric angle and relieving the duodenal compression.

Operative management, primarily laparoscopic duodenojejunostomy, is indicated only when nonoperative measures fail after an adequate trial (often suggested to be 6 weeks) or in the event of bowel necrosis. The rapid and complete recovery of our patient with conservative treatment, despite the severity of the initial complications, underscores the effectiveness of nutritional support in treating SMA syndrome.

## Conclusion

This case demonstrates that the presence of portal venous gas and gastric emphysema in SMA syndrome, which typically raises suspicion for gastrointestinal necrosis, can be successfully managed conservatively if clinical signs of perforation or sepsis are absent. Clinicians should maintain a high index of suspicion for the complex presentations of SMA syndrome and prioritize aggressive nonoperative nutritional support, as it can resolve obstruction and preclude the need for surgery even in the setting of severe multi-systemic complications.

## Data Availability

The datasets used during the current study are available from the corresponding author on reasonable request.
